# Regulatory miRNA–mRNA Networks in Parkinson’s Disease

**DOI:** 10.3390/cells10061410

**Published:** 2021-06-06

**Authors:** Bruno Lopes Santos-Lobato, Amanda Ferreira Vidal, Ândrea Ribeiro-dos-Santos

**Affiliations:** 1 Laboratório de Neuropatologia Experimental, Universidade Federal do Pará, Belém 66073-000, PA, Brazil; 2 Núcleo de Pesquisa em Oncologia, Programa de Pós-Graduação em Oncologia e Ciências Médicas, Universidade Federal do Pará, Belém 66073-000, PA, Brazil; amandaferreiravidal@gmail.com (A.F.V.); akelyufpa@gmail.com (Â.R.-d.-S.); 3 Instituto Tecnológico Vale, Belém 66055-090, PA, Brazil; 4 Laboratório de Genética Humana e Médica, Programa de Pós-Graduação em Genética e Biologia Molecular, Universidade Federal do Pará, Belém 66075-110, PA, Brazil

**Keywords:** Parkinson’s disease, microRNA, differentially expressed, neuronal survival signaling

## Abstract

Parkinson’s disease (PD) is the second-most common neurodegenerative disease, and its pathophysiology is associated with alpha-synuclein accumulation, oxidative stress, mitochondrial dysfunction, and neuroinflammation. MicroRNAs are small non-coding RNAs that regulate gene expression, and many previous studies have described their dysregulation in plasma, CSF, and in the brain of patients with PD. In this study, we aimed to provide a regulatory network analysis on differentially expressed miRNAs in the brain of patients with PD. Based on our systematic review with a focus on the substantia nigra and the putamen, we found 99 differentially expressed miRNAs in brain samples from patients with PD, which regulate 135 target genes. Five genes associated with neuronal survival (*BCL2*, *CCND1*, *FOXO3*, *MYC*, and *SIRT1*) were modulated by dysregulated miRNAs found in the substantia nigra and the putamen of patients with PD. The functional enrichment analysis found FoxO and PI3K-AKT signaling as pathways related to PD. In conclusion, our comprehensive analysis of brain-related miRNA–mRNA regulatory networks in PD showed that mechanisms involving neuronal survival signaling, such as cell cycle control and regulation of autophagy/apoptosis, may be crucial for the neurodegeneration of PD, being a promising way for novel disease-modifying therapies.

## 1. Introduction

Parkinson’s disease (PD) is the second-most common neurodegenerative disease, affecting approximately 6 million individuals worldwide, with a growing incidence in the last few decades [1]. Furthermore, the disease reduces life expectancy and increases disability-adjusted life years, and apparently, these negative impacts have not been mitigated by the advance of new therapies [2]. Some authors compare the recent expansion of PD to a pandemic and suggest a substantial increase in the funding of new research on its pathophysiology [1].

Multiple mechanisms are associated with the pathophysiology of PD, such as the accumulation of α-synuclein, mitochondrial dysfunction, oxidative stress, calcium homeostasis, and neuroinflammation [3]. These epigenetic mechanisms are influenced by microRNAs (miRNAs), small non-coding RNAs that regulate gene expression at a posttranscriptional level by binding to their target messenger RNAs (mRNAs) [4]. Several studies analyzed the differentially expressed miRNAs in biological samples from patients with PD; however, the low sample size and high methodological heterogeneity compromise the interpretation of these combined results [5]. A recent meta-analysis on miRNAs in PD identified 13 miRNAs that are consistently differentially expressed in the blood and brain of patients with PD, such as hsa-miR-133b, hsa-miR-221-3p, and hsa-miR-214-3p [5].

For instance, it was demonstrated that hsa-miR-34b and hsa-miR-34c are downregulated in PD, reducing the levels of DJ-1 and Parkin in the brain, two proteins involved in the ubiquitin–proteasome system in neurons, causing cell death. Furthermore, hsa-miR-4639-5p is upregulated in PD and inhibits DJ-1, also promoting cell death [6]. The dysregulation of these miRNAs shows how these molecules can modulate the pathophysiology of PD.

For a better understanding of the role of these miRNAs in PD pathophysiology, regulatory miRNA–mRNA networks, followed by their topological analysis and functional enrichment of the hub genes, are important to provide a broad view of the PD-related biological processes and signaling pathways [7,8]. The objective of this study was to explore PD pathophysiology through regulatory network analyses based on differentially expressed miRNAs (DE-miRNAs) in the brain of patients with PD described in previous studies, with a special focus on substantia nigra and putamen. These data can be useful for proposing miRNA-based therapies capable of slowing disease progression [9].

## 2. Materials and Methods

### 2.1. Screening of Candidates Differentially Expressed Brain-Related miRNAs Based on a Systematic Review

To screen differentially expressed miRNAs (DE-miRNAs) in the brain of patients with PD, we conducted a systematic literature search on MEDLINE, EMBASE, and Web of Science (from inception to December 2020) using the following algorithms: MEDLINE—“Parkinson’s disease” AND “microRNA” AND “brain”; EMBASE—(“Parkinson disease”/exp OR “Parkinson disease”) AND (“microrna”/exp OR “microRNA”) AND (“brain”/exp OR “brain”); Web of Science—ALL = (“Parkinson’s disease” AND “microRNA” AND “brain”). Reference lists of the studies included were checked to identify new studies missed in the primary search (cross-reference search).

### 2.2. Study Selection and Data Extraction

We aimed to select all original research studies describing DE-miRNAs in the brain of patients with PD. Two rounds of selection were performed. In the first round, titles and abstracts were screened and filtered following these exclusion criteria: (1) studies not conducted in patients with PD, (2) studies not conducted in human subjects, and (3) duplicate articles. In the second round, full texts were evaluated and excluded following other exclusion criteria: (1) review studies, (2) studies assessing different conditions from PD (such as atypical parkinsonism and dementia with Lewy bodies), (3) conference abstracts, and (4) full text not found. A single reviewer performed each selection round.

We extracted the following data: (1) the first author’s name, (2) year of publication, (3) brain region, (4) sample size, sex, and age of the study population (patients and controls), (5) dysregulated DE-miRNAs associated with PD, and (6) DE-miRNAs up- or downregulation in PD.

### 2.3. Prediction of the Target Genes of the Differentially Expressed Brain-Related miRNAs

After that, we predicted the target genes of the DE-miRNAs using miRTargetLink (https://ccb-web.cs.uni-saarland.de/mirtargetlink/ accessed on 7 January 2021), a tool for automating miRNA-targeting gene analysis procedures [10], considering only the strong evidence type of experimental validation. To filter the target genes, we downloaded RNA-Seq data from GTEx (https://gtexportal.org/home/ accessed on 7 January 2021) and selected only those that presented median gene-level TPM > 1 in all brain tissues (amygdala, anterior cingulate cortex, caudate—basal ganglia, cerebellar hemisphere, cerebellum, cortex, frontal cortex, hippocampus, hypothalamus, nucleus accumbens—basal ganglia, putamen, spinal cord, and substantia nigra). The filtered target genes were used in the following analyses.

### 2.4. Regulatory Networks and Their Topology Analysis

Regulatory networks of miRNA–mRNA interactions were constructed and visualized using Cytoscape software version 3.8.0 (http://www.cytoscape.org/ (accessed on 7 January 2021) [11]. We analyzed the networks’ centrality (degree, betweenness, and closeness) and identified the hub genes using the CytoNCA plugin [12] in Cytoscape [13]. Hub gene expressions in GTEx brain tissues were plotted in heatmaps using the *pheatmap* package in R (Version 1.2.5033).

### 2.5. Functional Enrichment Analysis

Functional enrichment analysis of the target genes was performed using *clusterProfiler* and *org.Hs.eg.db* packages in R (Version 1.2.5033) [14]. The enriched Kyoto Encyclopedia of Genes and Genomes (KEGG) pathways were plotted using the *clusterProfiler* package in R (Version 1.2.5033).

## 3. Results

### 3.1. Differentially Expressed Brain-Related miRNAs Based on the Systematic Review

After pooling the publications from the databases, a total of 880 publications were found. After both rounds of selection, a total of 19 articles were finally included and reviewed (Table 1) [14,15,16,17,18,19,20,21,22,23,24,25,26,27,28,29,30,31,32]. Among these studies, the most collected brain regions were the substantia nigra (*n* = 9), neocortical areas (*n* = 9, including prefrontal, frontal, anterior cingulate and temporal cortex), putamen (*n* = 2), amygdala (*n* = 2) and cerebellum (*n* = 2). All samples were extracted from postmortem brains, and the median postmortem interval (PMI) was higher than 12 h (PD: median PMI 18.35 h, interquartile range [IQR] 10–49; controls: median PMI 23.95 h, IQR 15–47). The sample size was less than 10 in most studies (PD: median number 8, IQR 6–15; controls: median number 8, IQR 5.5–11.5), and most participants died over 65 years (PD: median age at death 76 years, IQR 71.5–77; controls: median age at death 69 years, IQR 68.5–74). The median disease duration of patients with PD was 8 years (IQR 5–12).

The number of brain-related DE-miRNAs associated with PD varied from 1 to 29 per study (median 1, IQR 1–8). A total of 99 brain-related DE-miRNAs associated with PD were reported by the selected studies: 60 upregulated miRNAs and 39 downregulated miRNAs—only hsa-miR-144 was reported as upregulated and downregulated in different studies (Table 2).

Particularly, we analyzed samples from substantia nigra and putamen, which comprise the nigrostriatal pathway, a brain circuit with relevant importance to PD. From the substantia nigra samples, 38 DE-miRNAs were reported, while 14 DE-miRNAs were reported related to putamen (Table 2). Two DE-miRNAs, hsa-miR-34b and hsa-miR-95, were dysregulated in both substantia nigra and putamen.

### 3.2. Analysis of the Differentially Expressed Brain-Related miRNAs’ Target Genes

**Figure 1:**
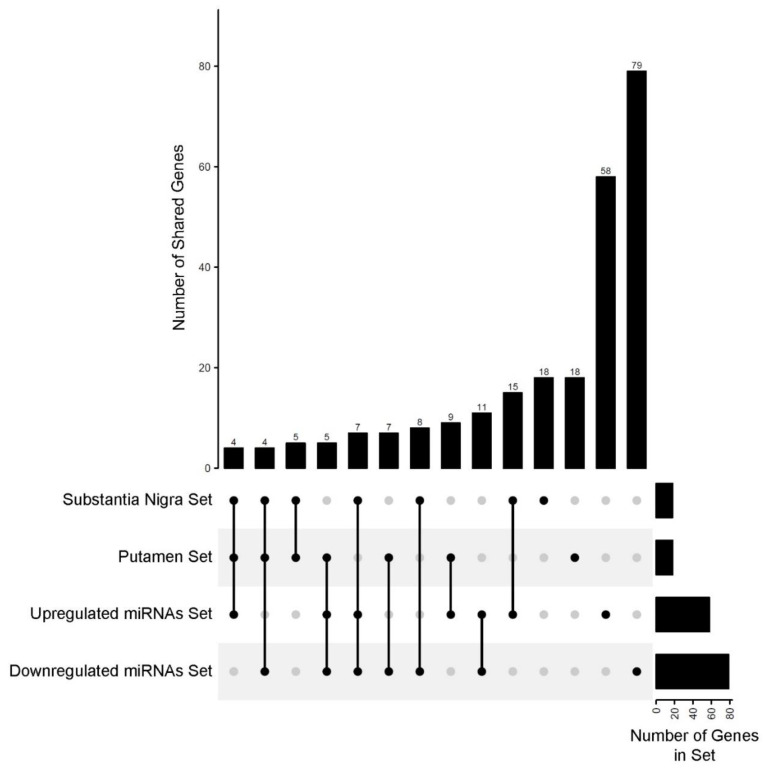
Number of shared target genes between the four sets of differentially expressed miRNAs (vertical bars). The lower part of the figure shows the intersection of sets associated with the vertical bars (dots connected by black lines).

Target genes prediction was performed using an experimentally validated microRNA–target interactions database. The predicted targets were filtered according to the GTEx data by considering only those that presented median gene-level TPM > 1 in the brain. This approach resulted in 58 target genes for the upregulated brain-related miRNAs and 79 genes for the downregulated miRNAs (Table 3). Especially for the DE-miRNAs found in the substantia nigra and putamen, we found 22 and 18 target genes, respectively (Table 3). When comparing these results, we found some common target genes between the four sets (Figure 1). For instance, we identified that three genes (*CCND1*, *FOXO3*, and *SIRT1*) are in common between all sets, while five genes are shared between substantia nigra and putamen (*BCL2*, *CCND1*, *FOXO3*, *MYC*, and *SIRT1*) (Table S1).

**Figure 2:**
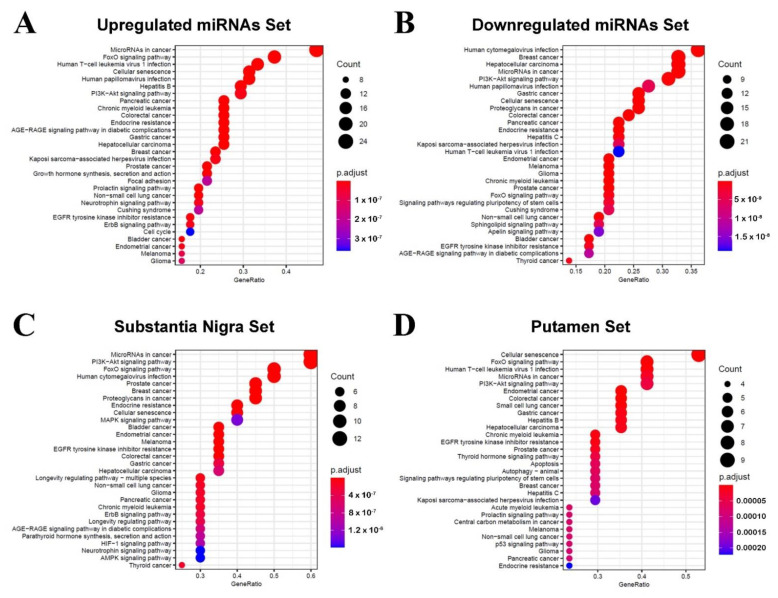
Enriched KEGG pathways for the target genes regulated by the four sets of differentially expressed miRNAs. (**A**) Upregulated DE-miRNAs’ target gene enrichment. (**B**) Downregulated DE-miRNAs’ target gene enrichment. (**C**) Substantia nigra DE-miRNAs’ target gene enrichment. (**D**) Putamen DE-miRNAs’ target genes enrichment. The color of the circles indicates the significance of the pathway, and the size of the circles indicates the number of target genes involved in each pathway.

After that, we performed a functional enrichment analysis for each gene set (Tables S2–S5) and plotted the 30 most significant KEGG pathways (Figure 2). Among the enriched KEGG pathways, we highlight FoxO and PI3K-AKT signaling pathways, which are processes closely related to PD.

### 3.3. Regulatory Networks and Their Topology Analysis

**Figure 3:**
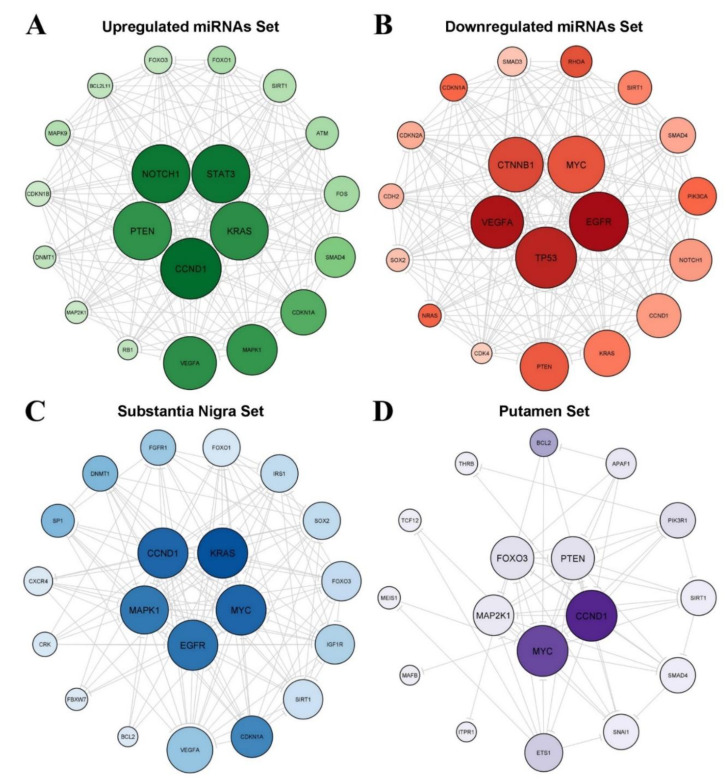
Regulatory networks for the four sets of differentially expressed miRNAs including the top 20 hub nodes. (**A**) Upregulated DE-miRNAs–mRNA network. (**B**) Downregulated DE-miRNAs–mRNA network. (**C**) Substantia nigra DE-miRNAs–mRNA network. (**D**) Putamen DE-miRNAs–mRNA network. The node size indicates degree centrality, and the scale of the node color indicates betweenness centrality. The center of the network displays the top five hub nodes according to degree centrality.

We constructed four miRNA–mRNA regulatory networks: (1) upregulated DE-miRNAs and their targets, resulting in 54 nodes and 439 interactions (Figure 3A); (2) downregulated DE-miRNAs and its targets, resulting in 73 nodes and 574 interactions (Figure 3B); (3) substantia nigra DE-miRNAs and their targets (Figure 3C), which involved 21 nodes and 126 interactions; and (4) putamen DE-miRNAs and their targets, represented by 17 nodes and 54 interactions (Figure 3D).

**Figure 4:**
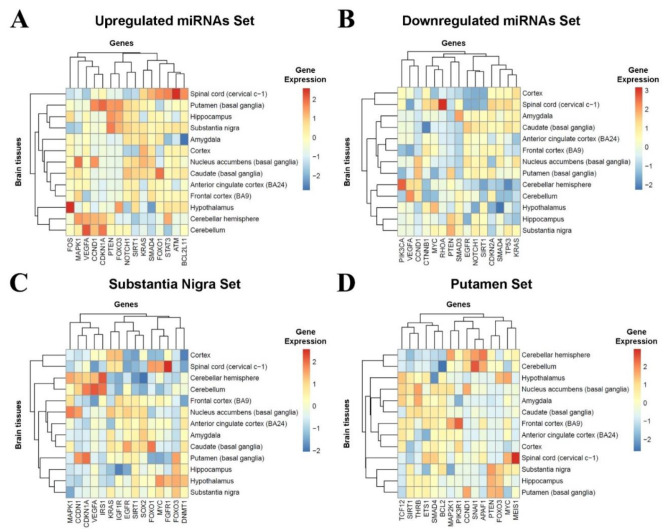
Heatmaps showing the hierarchical clustering of the global expression of the target genes regulated by the four sets of differentially expressed miRNAs across human brain tissues from the Genotype-Tissue Expression. (**A**) Upregulated DE-miRNAs’ hub genes expression. (**B**) Downregulated DE-miRNAs’ hub genes expression. (**C**) Substantia nigra DE-miRNAs’ hub gene expression. (**D**) Putamen DE-miRNAs’ hub gene expression.

As shown in Figure 3, some genes potentially have a central role in the regulatory networks, such as *CCND1* and *MYC*. To better identify these hub genes, we analyzed the degree, betweenness, and closeness centrality of the nodes (Table 4). After identifying the hub genes of each network, we analyzed their expression across the brain regions using GTEx RNA-Seq data (Figure 4). Overall heatmaps evidence the high expression of *PTEN* and *CCND1* in substantia nigra and putamen, respectively, suggesting the potential role of these genes in the brain.

## 4. Discussion

Based on a systematic review, we found a total of 99 DE-miRNAs (including 60 upregulated miRNAs and 39 downregulated miRNAs) in brain samples from patients with PD compared to healthy controls. Among them, hsa-miR-144 is the only one found as both up- and downregulated in PD—there is some evidence showing that this miRNA modifies the expression of three genes associated with monogenic forms of PD (*SNCA*, *PRKN*, *LRRK2*) [27]. Cho et al. showed that an inverse correlation between hsa-miR-205 and *LRRK2* in PD was previously described, with high LRRK2 protein expression and low hsa-miR-205 levels in the frontal cortex of patients with PD, probably due to the 3′-UTR region of *LRRK2* being an hsa-miR-205 target site [18]. A former study showed that hsa-miR-7, which was downregulated in the substantia nigra according to our review, is a direct regulator of *SNCA*, reducing its expression in a cell model and in an MPTP PD murine model [34]. Considering the miRNAs associated with both substantia nigra and putamen, we found that hsa-miR-34b and hsa-miR-95-hsa-miR-34b are associated with a reduction in the expression of alpha-synuclein [35], DJ-1, and Parkin [6], while hsa-miR-95 regulates the lysosomal function through the enzyme sulfatase-modifying factor 1 [36], and it was downregulated in pregnant women with multiple sclerosis [37]. To compare with another prevalent neurodegenerative disease, miRNAs such as hsa-miR-132 and hsa-miR-339-5p could be found in the brain of both PD and Alzheimer’s disease patients [38].

Together, the DE-miRNAs regulate 135 genes. From these, five genes are regulated simultaneously by the dysregulated sets of miRNAs found in the substantia nigra and the putamen of patients with PD (*BCL2*, *CCND1*, *FOXO3*, *MYC*, and *SIRT1*) (Table 4 and Figure 3). These genes have central roles in the miRNA–mRNA regulatory networks, and some of them have high expression in the brain, particularly in the substantia nigra and the putamen (Figure 3 and Figure 4).

Cyclin D1 (CCND1) is a regulator of the cell cycle progression mediated by extracellular stimulation, and its overexpression results in neoplastic growth [39], or apoptotic-related cell death in postmitotic neurons [40]. The re-expression of cyclins and cyclin-dependent kinases in neurons from patients with Alzheimer’s disease suggests that the failure of cell cycle arrest in adults may be associated with late-onset neurodegenerative diseases [40]. In PD, there is an overexpression of mitotic-associated proteins, such as cyclins and cyclin-dependent kinases, in the substantia nigra of postmortem patients with PD and an MPTP mouse model of PD, resulting in apoptosis of dopaminergic neurons [41,42]. Recently, some cell cycle genes were found enriched in a cell model of PD, and CCND1 was reported as upregulated and involved in alpha-synuclein cell death. It was shown that the knockdown of CCND1 reduces cell death [43], reinforcing our results of upregulation of miRNAs that regulate CCND1 in the brain.

Forkhead box protein O3 (FOXO3), comprising the Forkhead family, is a transcription factor associated with longevity in humans, and it is expressed in dopaminergic neurons of the substantia nigra. In a rat model of PD, FOXO3 was essential in the neuronal survival of the substantia nigra, and it may also reduce alpha-synuclein accumulation and its toxicity [44]. Also extending longevity, the silence information regulator 1 (SIRT1) is a member of the sirtuin family, which regulates DNA stability and controls gene expression and cell cycle progression. Enzymatic activity of *SIRT1* is reduced in the temporal and frontal cortex of patients with PD [45], playing a critical role in the pathophysiology of PD through induction of autophagy, regulation of mitochondrial function, inhibition of neuroinflammation, and increasing degradation of alpha-synuclein [46].

*MYC* (or c-myc) is a transcription factor that regulates cell growth, division, differentiation, and death, and despite having a classic role in brain cancer progression and brain development, *MYC* expression is increased in neurodegenerative diseases, such as Alzheimer’s disease, and like CCND1, its role is based on cell cycle control [47]. *BCL2* is a suppressor of autophagy and apoptotic cell death, and its expression is decreased in cell models of PD [48].

Despite being mostly related to cancer, two of the pathways associated with PD are closely associated with neuronal survival and neurodegenerative diseases: FoxO and PI3K-AKT signaling pathways. The Forkhead box class O (FoxO) family of transcription factors has an essential role in multiple cellular processes in the nervous system, such as neural development and neuronal survival, promoting a proapoptotic effect [49]; otherwise, the PI3K-AKT pathway is associated with neuroprotection and is a major regulator of the FoxO pathway, inhibiting FoxO-induced neuronal death [49].

Previous studies have explored pathways involved in PD pathogenesis. Song et al. [50] found 21 different pathways associated with PD, based on GWAS datasets. In another study, data from Gene Expression Omnibus from patients with PD were used to perform regulatory network and functional and enrichment analysis, showing that distinct pathways, such as amoebiasis and MAPK signaling, might be related to PD [51]. More recently, another study also used a dataset from Gene Expression Omnibus and revealed new 12 pathways associated with PD [52].

These results suggest that, in PD, the expression of genes involved in cell survival is dysregulated by miRNAs. Therefore, besides alpha-synuclein accumulation, oxidative stress, and neuroinflammation, the neurodegeneration of PD may include competing mechanisms over neuronal survival, such as cell cycle control and regulation of autophagy/apoptosis, particularly in the substantia nigra. Neuronal survival signaling may become the target of new disease-modifying treatments for PD, including the use of miRNA-based therapies [9].

## 5. Conclusions

In conclusion, our analysis of miRNAs associated with PD based on a systematic review showed a multitude of differentially expressed genes in the brain of these patients, especially in the substantia nigra. This expression dysregulation is linked to several pathways, including neuronal survival signaling. The role of these genes and pathways must be explored in further studies and can be used by future studies on miRNA-based therapies.

## Figures and Tables

**Table 1:** The main characteristics of 19 studies involving brain-related differentially expressed miRNA in Parkinson’s disease.

Author, Year	Country	Brain Region	Sample Size	Age at Death	Disease Duration (years)	Postmortem Interval (hours)	PD Braak Staging	miRNAs	Upreg miRNAs	Downreg miRNAs
Kim et al., 2007 [15]	USA	Midbrain, cerebellum, frontal and prefrontal cortex	3	70	NA	NA	NA	1	0	1
Sethi and Lukiw, 2009 [16]	USA	Temporal cortex	4	69	NA	1.2	NA	0	0	0
Miñones-Moyano et al., 2011 [17]	Spain	SN, amygdala, cerebellum, frontal cortex	14	72	NA	6.4	4	2	0	2
Cho et al., 2013 [18]	USA	Frontal cortex	15	80	NA	8.2	3.5	1	0	1
Alvarez-Erviti et al., 2013 [19]	Spain	SN, amygdala	6	76	NA	4.8	NA	6	6	0
Kim et al., 2014 [20]	USA	SN	8	78	NA	20.7	NA	1	1	0
Schlaudraff et al., 2014 [21]	Germany	SN	5	78	NA	16	NA	0	0	0
Villar-Menéndez et al., 2014 [22]	Spain	Putamen	6	76	NA	7.9	4	1	0	1
Cardo et al., 2014 [23]	UK	SN	8	77	4,25	45.8	NA	10	9	1
Briggs et al., 2015 [24]	USA	SN	8	NA	NA	NA	NA	17	15	2
Pantano et al., 2015 [25]	Spain	Amygdala	7	70	NA	NA	NA	0	0	0
Wake et al., 2016 [26]	USA	Prefrontal cortex	29	77	NA	8	NA	0	0	0
Tatura et al., 2016 [27]	Germany	Anterior cingulate cortex	22	73	NA	30.6	NA	5	5	0
Nair and Ge, 2016 [28]	USA	Putamen	12	75	NA	13.4	NA	13	6	7
Hoss et al., 2016 [29]	USA	Prefrontal cortex	29	77	10,5	11.1	NA	29	11	18
Chatterjee and Roy, 2017 [30]	India	Prefrontal cortex	29	NA	NA	NA	NA	11	9	2
McMillan et al., 2017 [31]	UK	SN	6	83	16,1	NA	NA	1	0	1
Xing et al., 2020 [32]	China	Prefrontal cortex	15	70	5,5	NA	NA	3	0	3
Hu et al., 2020 [33]	China	SN	4	NA	NA	NA	NA	1	0	1

Abbreviations: Downreg miRNAs, number of downregulated miRNAs described by the study; miRNAs, number of differentially expressed miRNAs described by the study; NA, not available; PD, Parkinson’s disease; SN, substantia nigra; Upreg miRNAs, number of upregulated miRNAs described by the study.

**Table 2:** Brain-related differentially expressed miRNAs in Parkinson’s disease described by previous studies.

Upregulated miRNAs	Downregulated miRNAs	SN DE-miRNAs	Putamen DE-miRNAs
hsa-let-7b	hsa-miR-10b-5p	hsa-miR-133b	hsa-miR-155-5p
hsa-let-7d-5p	hsa-miR-124	hsa-miR-34b	hsa-miR-219-2-3p
hsa-let-7f-5p	hsa-miR-1294	hsa-miR-34c	hsa-miR-3200-3p
hsa-miR-106a ^§^	hsa-miR-129-5p	hsa-miR-425	hsa-miR-34b
hsa-miR-106b-5p	hsa-miR-132-3p	hsa-miR-532-5p	hsa-miR-382-5p
hsa-miR-126	hsa-miR-132-5p	hsa-miR-548d	hsa-miR-421
hsa-miR-132	hsa-miR-133b	hsa-miR-7	hsa-miR-423-5p
hsa-miR-135a	hsa-miR-144	hsa-miR-774	hsa-miR-4421
hsa-miR-135b	hsa-miR-145-5p	hsa-let-7b	hsa-miR-204-5p
hsa-miR-144	hsa-miR-148b-3p	hsa-miR-106a ^§^	hsa-miR-221-3p
hsa-miR-144-3p	hsa-miR-155-5p	hsa-miR-126	hsa-miR-3195
hsa-miR-144-5p	hsa-miR-205	hsa-miR-132	hsa-miR-425-5p
hsa-miR-145	hsa-miR-212-5p	hsa-miR-135a	hsa-miR-485-3p
hsa-miR-148a	hsa-miR-217	hsa-miR-135b	hsa-miR-95
hsa-miR-151b	hsa-miR-218	hsa-miR-145	
hsa-miR-15b-5p	hsa-miR-219-2-3p	hsa-miR-148a	
hsa-miR-16-2-3p	hsa-miR-3200-3p	hsa-miR-184	
hsa-miR-181a-5p	hsa-miR-320b	hsa-miR-198	
hsa-miR-184	hsa-miR-324-5p	hsa-miR-208b	
hsa-miR-198	hsa-miR-338-5p	hsa-miR-21 *	
hsa-miR-199b	hsa-miR-34b ^§^	hsa-miR-223	
hsa-miR-204-5p	hsa-miR-34c	hsa-miR-224	
hsa-miR-208b	hsa-miR-362-5p	hsa-miR-26a	
hsa-miR-21 *	hsa-miR-378c	hsa-miR-26b	
hsa-miR-216b-5p	hsa-miR-380-5p	hsa-miR-27a	
hsa-miR-221	hsa-miR-382-5p	hsa-miR-28-5p	
hsa-miR-221-3p	hsa-miR-421	hsa-miR-299-5p	
hsa-miR-223	hsa-miR-423-5p	hsa-miR-301b	
hsa-miR-224	hsa-miR-425	hsa-miR-330-5p	
hsa-miR-26a	hsa-miR-4421	hsa-miR-335	
hsa-miR-26b	hsa-miR-490-5p	hsa-miR-337-5p	
hsa-miR-27a	hsa-miR-491-5p	hsa-miR-339-5p	
hsa-miR-28-5p	hsa-miR-532-5p	hsa-miR-373 *	
hsa-miR-299-5p	hsa-miR-548d	hsa-miR-374a	
hsa-miR-301b	hsa-miR-6511a-5p	hsa-miR-485-5p	
hsa-miR-3117-3p	hsa-miR-670-3p	hsa-miR-542-3p	
hsa-miR-3195	hsa-miR-671-5p	hsa-miR-92a	
hsa-miR-330-5p	hsa-miR-7	hsa-miR-95	
hsa-miR-335	hsa-miR-774		
hsa-miR-337-5p			
hsa-miR-339-5p			
hsa-miR-373 *			
hsa-miR-374a			
hsa-miR-376c-5p			
hsa-miR-425-5p			
hsa-miR-4443			
hsa-miR-454-3p			
hsa-miR-485-3p			
hsa-miR-485-5p			
hsa-miR-488			
hsa-miR-5100			
hsa-miR-516b-5p			
hsa-miR-542-3p			
hsa-miR-544			
hsa-miR-5690			
hsa-miR-92a			
hsa-miR-92a-3p			
hsa-miR-92b-3p			
hsa-miR-93-5p			
hsa-miR-95 ^§^			

Abbreviations: ^§^, miRNA described in more than one study; SN DE-miRNAs, miRNAs differentially expressed in substantia nigra; Putamen DE-miRNAs, miRNAs differentially expressed in putamen. miRNAs in green indicate upregulated miRNAs, and red indicate downregulated miRNAs.

**Table 3:** Predicted genes targeted by brain-related differentially expressed miRNAs in Parkinson’s disease.

For Upreg miRNAs	For Downreg miRNAs	For SN DE-miRNAs	For Putamen DE-miRNAs
*APC*	*ADD3*	*BCL2*	*APAF1*
*APP*	*ANXA2*	*CCND1*	*BCL2*
*ATG16L1*	*APC*	*CDKN1A*	*CCND1*
*ATM*	*ARID2*	*CRK*	*ETS1*
*BCL2*	*ARL6IP5*	*CXCR4*	*FOXO3*
*BCL2L11*	*CAMTA1*	*DNMT1*	*ITPR1*
*CCND1*	*CBFB*	*EGFR*	*MAFB*
*CDKN1A*	*CCND1*	*FBXW7*	*MAP2K1*
*CDKN1B*	*CDH2*	*FGFR1*	*MEIS1*
*CDKN1C*	*CDK4*	*FOXO1*	*MYC*
*CRK*	*CDK6*	*FOXO3*	*PIK3R1*
*DDIT4*	*CDKN1A*	*IGF1R*	*PTEN*
*DICER1*	*CEBPA*	*IRS1*	*SIRT1*
*DNMT1*	*CHRAC1*	*KRAS*	*SMAD4*
*E2F1*	*CPNE3*	*MAPK1*	*SNAI1*
*E2F5*	*CSRP1*	*MYC*	*SSX2IP*
*EZR*	*CTGF*	*PTBP2*	*TCF12*
*FBXW7*	*CTNNB1*	*SIRT1*	*THRB*
*FOS*	*DDX6*	*SOX2*	
*FOXO1*	*DNAJB1*	*SP1*	
*FOXO3*	*E2F3*	*SP3*	
*HIPK2*	*EDN1*	*VEGFA*	
*IRS1*	*EGFR*		
*ITGA5*	*EIF4E*		
*ITGB8*	*ERG*		
*KAT2B*	*ETS1*		
*KRAS*	*FLI1*		
*MAFB*	*FLOT2*		
*MAP2K1*	*FOXO3*		
*MAP2K4*	*FSCN1*		
*MAPK1*	*FZD7*		
*MAPK9*	*GNA13*		
*NFE2L2*	*GNAI2*		
*NLK*	*GNAI3*		
*NOTCH1*	*GOLGA7*		
*NTRK3*	*HCN2*		
*PTEN*	*IGF1R*		
*PURA*	*IL6R*		
*RAP1B*	*JAG1*		
*RB1*	*JUP*		
*RECK*	*KLF4*		
*RGS5*	*KRAS*		
*SIRT1*	*LIN7C*		
*SMAD4*	*LRP1*		
*SMAD7*	*MECP2*		
*SOCS3*	*MEF2A*		
*SP1*	*MYC*		
*SP3*	*NOTCH1*		
*STAT3*	*NRAS*		
*STAT5A*	*NT5E*		
*TCEAL1*	*PDLIM7*		
*TCF4*	*PHC2*		
*TGFBR1*	*PICALM*		
*TGFBR2*	*PIK3CA*		
*THRB*	*PODXL*		
*TMED7*	*PSIP1*		
*VEGFA*	*PSMG1*		
*ZBTB4*	*PTBP1*		
	*PTBP2*		
	*PTEN*		
	*RAB11FIP2*		
	*RAC1*		
	*RHOA*		
	*ROCK1*		
	*SIRT1*		
	*SMAD3*		
	*SMAD4*		
	*SOX2*		
	*SOX9*		
	*SP1*		
	*SWAP70*		
	*SYNE1*		
	*TAGLN2*		
	*TP53*		
	*TPM1*		
	*TPM3*		
	*TWF1*		
	*VEGFA*		
	*YWHAZ*		

Abbreviations: DE-miRNAs, differentially expressed miRNA; Downreg; downregulated; SN; substantia nigra; Upreg, upregulated.

**Table 4:** The top 15 hub nodes in the regulatory networks associated with brain-related differentially expressed miRNAs in Parkinson’s disease.

**Regulatory Network Targeted by Upregulated miRNAs**						**Regulatory Network Targeted by Downregulated miRNAs**					
**Node**	**DC**	**Node**	**BC**	**Node**	**CC**	**Node**	**DC**	**Node**	**BC**	**Node**	**CC**
*CCND1*	37	*CCND1*	199.83435	*CCND1*	0.7571428	*TP53*	44	*EGFR*	648.0353	*TP53*	0.6923077
*STAT3*	36	*NOTCH1*	188.92195	*NOTCH1*	0.7571428	*EGFR*	43	*VEGFA*	606.03754	*EGFR*	0.6857143
*PTEN*	36	*STAT3*	188.05045	*PTEN*	0.7571428	*MYC*	42	*TP53*	524.7005	*MYC*	0.6792453
*NOTCH1*	36	*KRAS*	162.67937	*KRAS*	0.7464788	*CTNNB1*	41	*CTNNB1*	334.24814	*VEGFA*	0.6666667
*KRAS*	36	*VEGFA*	162.09471	*STAT3*	0.7464788	*VEGFA*	40	*RHOA*	282.5179	*PTEN*	0.6605505
*VEGFA*	34	*PTEN*	157.23015	*VEGFA*	0.7361111	*PTEN*	38	*MYC*	268.5208	*CTNNB1*	0.6545454
*MAPK1*	33	*MAPK1*	153.92905	*MAPK1*	0.7162162	*KRAS*	37	*ANXA2*	264.8916	*KRAS*	0.6371681
*CDKN1A*	31	*CDKN1A*	126.64957	*CDKN1A*	0.6973684	*CCND1*	36	*PTEN*	236.95053	*CCND1*	0.6260869
*SMAD4*	29	*E2F1*	111.34685	*SMAD4*	0.6794871	*NOTCH1*	35	*NRAS*	180.74113	*NOTCH1*	0.6206896
*FOS*	27	*SMAD4*	90.59089	*FOS*	0.654321	*PIK3CA*	33	*PIK3CA*	167.35359	*PIK3CA*	0.6153846
*ATM*	26	*CRK*	57.47987	*SIRT1*	0.654321	*SMAD4*	32	*CDKN1A*	166.20766	*SMAD4*	0.6
*SIRT1*	26	*FOS*	57.051147	*ATM*	0.654321	*SIRT1*	31	*TPM1*	154.96588	*RHOA*	0.6
*FOXO1*	24	*ATM*	52.87451	*FOXO1*	0.6385542	*RHOA*	30	*TAGLN2*	144.89015	*SIRT1*	0.5901639
*FOXO3*	24	*TGFBR1*	52.30357	*CDKN1B*	0.6309523	*SMAD3*	29	*PSIP1*	142.28922	*CDKN1A*	0.5853658
*BCL2L11*	23	*FOXO1*	49.671513	*FOXO3*	0.6309523	*CDKN2A*	28	*FLI1*	142.0	*CDKN2A*	0.5806451
**Regulatory Network Targeted by DE-miRNAs in SN**						**Regulatory Network Targeted by DE-miRNAs in Putamen**					
**Node**	**DC**	**Node**	**BC**	**Node**	**CC**	**Node**	**DC**	**Node**	**BC**	**Node**	**CC**
*EGFR*	18	*KRAS*	24.592207	*CCND1*	0.9090909	*MYC*	13	*CCND1*	69.53333	*CCND1*	0.8421052
*MYC*	18	*CCND1*	21.90339	*KRAS*	0.9090909	*CCND1*	13	*MYC*	59.533333	*MYC*	0.8421052
*KRAS*	18	*MYC*	21.90339	*MYC*	0.9090909	*PTEN*	10	*BCL2*	30.0	*FOXO3*	0.6956522
*CCND1*	18	*EGFR*	20.09127	*EGFR*	0.9090909	*FOXO3*	10	*ETS1*	14.333333	*PTEN*	0.6956522
*MAPK1*	17	*MAPK1*	19.217676	*MAPK1*	0.8695652	*MAP2K1*	9	*PIK3R1*	6.3333335	*MAP2K1*	0.6666667
*VEGFA*	16	*CDKN1A*	17.548702	*VEGFA*	0.8333333	*ETS1*	8	*FOXO3*	5.2	*ETS1*	0.64
*CDKN1A*	14	*DNMT1*	10.332828	*CDKN1A*	0.7692308	*SNAI1*	7	*PTEN*	5.2	*PIK3R1*	0.6153846
*SIRT1*	13	*SP1*	10.186725	*FOXO3*	0.7407407	*SMAD4*	7	*APAF1*	2.6666667	*SIRT1*	0.6153846
*IGF1R*	13	*VEGFA*	7.7579365	*IGF1R*	0.7407407	*SIRT1*	7	*MAP2K1*	2.2	*SMAD4*	0.6153846
*FOXO3*	13	*FGFR1*	6.8968253	*SIRT1*	0.7407407	*PIK3R1*	7	*SIRT1*	0.3333333	*SNAI1*	0.6153846
*SOX2*	12	*IGF1R*	5.0380955	*FOXO1*	0.7142857	*APAF1*	5	*SMAD4*	0.3333333	*APAF1*	0.5925926
*IRS1*	12	*IRS1*	3.0833333	*SOX2*	0.7142857	*BCL2*	4	*SNAI1*	0.3333333	*BCL2*	0.5714286
*FOXO1*	12	*SOX2*	3.0269842	*DNMT1*	0.6896552	*THRB*	2	*ITPR1*	0.0	*MEIS1*	0.4848485
*FGFR1*	11	*FOXO3*	2.8960319	*FGFR1*	0.6896552	*TCF12*	2	*MAFB*	0.0	*TCF12*	0.4848485
*DNMT1*	11	*SIRT1*	1.9690477	*IRS1*	0.6896552	*MEIS1*	2	*MEIS1*	0.0	*THRB*	0.4848485

Abbreviations: BC, betweenness centrality; CC, closeness centrality; DC, degree centrality; DE-miRNAs, differentially expressed miRNA; SN; substantia nigra.

## Data Availability

All data are included in the paper.

## Supplementary Materials

The following are available online at https://www.mdpi.com/article/10.3390/cells10061410/s1: Table S1: Target genes in common between the four sets of miRNAs (upregulated miRNAs, downregulated miRNAs, substantia nigra, and putamen); Table S2: KEGG pathways enrichment for the upregulated DE-miRNAs’ target genes; Table S3: KEGG pathways enrichment for the downregulated DE-miRNAs’ target genes; Table S4: KEGG pathways enrichment for the substantia nigra DE-miRNAs’ target genes; Table S5: KEGG pathways enrichment for the putamen DE-miRNAs’ target genes.
